# Predictive Factors for Recovery Time in Conceived Women Suffering From Moderate to Severe Ovarian Hyperstimulation Syndrome

**DOI:** 10.3389/fendo.2022.870008

**Published:** 2022-06-15

**Authors:** Kai Huang, Ying Shi, Gezi Chen, Hao Shi, Jun Zhai

**Affiliations:** ^1^Center for Reproductive Medicine, Henan Key Laboratory of Reproduction and Genetics, Henan Provincial Obstetrical and Gynecological Diseases (Reproductive Medicine) Clinical Research Center, Henan Engineering Laboratory of Preimplantation Genetic Diagnosis and Screening, The First Affiliated Hospital of Zhengzhou University, Zhengzhou, China; ^2^Department of Obstetrics, The First Affiliated Hospital of Zhengzhou University, Zhengzhou, China

**Keywords:** ovarian hyperstimulation syndrome (OHSS), clinical features, recovery time, potential predictors, coagulation function

## Abstract

**Objective:**

This study aimed to evaluate potential predictors for recovery time in pregnant patients with moderate to severe ovarian hyperstimulation syndrome (OHSS).

**Methods:**

A total of 424 pregnant patients with moderate to severe OHSS who underwent *in vitro* fertilization (IVF)/intracytoplasmic sperm injection (ICSI) were retrospectively identified. The clinical features and laboratory findings within 24 h after admission were collected. Treatment for OHSS was carried out according to standard procedures, including fluid replacement therapy, human albumin, aspirin, low-molecular-weight heparin, and paracentesis, when necessary. Patients were discharged from the hospital when the tmorning hematocrit was <40% and no obvious clinically relevant symptoms existed, such as abdominal distension, abdominal pain, and shortness of breath. Meanwhile, ultrasound indicating little pleural or abdominal effusion and biochemical abnormalities returning to normal were required. Spearman’s correlation analysis was used to assess the association between the blood-related parameters and recovery time. Multiple linear regression models were used to assess the relationship between the clinical or laboratory parameters and recovery time.

**Results:**

The median recovery time of these patients was 11 days. In Spearman’s correlation test, leukocytes, hemoglobin, platelets, hematocrit, creatinine, prothrombin time (PT), fibrinogen (Fib), D-dimer, and fibrinogen degradation products (FDPs) were positively correlated with recovery time. On the other hand, albumin and thrombin time (TT) were negatively correlated with recovery time. Multiple linear regression analysis showed that polycystic ovary syndrome (PCOS), hemoglobin, platelets, albumin, and Fib were significantly associated with the recovery time of patients with OHSS (*p* = 0.023, *p* < 0.001, *p* = 0.007, *p* < 0.001, and *p* = 0.019, respectively).

**Conclusions:**

In pregnant patients with OHSS, PCOS and hypoalbuminemia were associated with a significantly longer recovery time. Meanwhile, the recovery time was longer when patients have high levels of hemoglobin, platelets, and Fib.

## Introduction

Ovarian hyperstimulation syndrome (OHSS) is a self-limiting disease classically encountered in patients who undergo controlled ovarian hyperstimulation (COH) cycles. The mild manifestations of OHSS include nausea, vomiting, abdominal distension, and shortness of breath. In severe cases, ascites and pleural fluid may occur, causing respiratory, circulatory, and coagulation dysfunction, and especially thrombosis may endanger a patient’s life (1).

Evidence from initial investigations indicates that some women are at increased risk of OHSS. The risk factors include young age (2, 3), body mass index (BMI) (4, 5), diagnosis of polycystic ovary syndrome (PCOS) (3, 6) high anti-müllerian hormone (AMH) levels (7, 8), large number and size of follicles in the ovary (8, 9), high serum estradiol (E2) concentrations (3, 9, 10) high number of retrieved oocytes (3, 9, 11), pregnancy following fresh embryo transfer (12), and a history of OHSS (13). The exact cause of OHSS is currently complex and remains subject to controversy. Latest research has demonstrated that OHSS is related to age, BMI, ovarian function, and the ovulation stimulation protocol.

Evidence has shown that OHSS occurs only after exposure to human chorionic gonadotropin (hCG), which has a significantly longer half-life than that of luteinizing hormone (LH) and a higher receptor affinity, thus causing extensive luteinization in the granulosa cells within the corpus luteum (14). This, in turn, leads to the production of vasoactive substances, including vascular endothelial growth factor (VEGF), renin–angiotensin system, interleukin 6, interleukin 1b, angiotensin II, insulin-like growth factor 1, and transforming growth factor b, of which VEGF is the most important in causing increased vascular permeability and hemoconcentration (15–17). VEGF stimulates endothelial cell mitogenesis and renders capillaries highly permeable to high-molecular-weight proteins (15). The pathophysiology of OHSS is characterized by arteriolar vasodilation and an increase in capillary permeability, leading to the leakage of fluid from the vascular compartment, with third space fluid accumulation and intravascular dehydration, causing intravascular volume depletion, hemoconcentration, hypoalbuminemia, electrolyte imbalance, and even thrombosis.

Whereas treatment for OHSS is largely supportive, prevention is crucial. Most current studies have been devoted to prevention and treatment strategies for OHSS, with relatively little attention paid to its clinical prognosis. This study provides clinicians with potential predictors of time to cure by describing some clinical features and laboratory findings in pregnant patients with OHSS.

## Materials and Methods

### Study Population

This retrospective study was performed in the Reproductive Medical Center of The First Affiliated Hospital of Zhengzhou University, Henan Province, China. Patients who underwent *in vitro* fertilization (IVF)/intracytoplasmic single sperm injection (ICSI) after assisted conception with late-onset moderate to severe OHSS between January 2018 and December 2020 were selected. The access and processing of patient data was approved by the ethics committee under a protocol for retrospective studies. The inclusion criteria were as follows: 1) diagnosis of OHSS according to the Golan criteria; 2) patients with IVF/ICSI-assisted pregnancy in the first cycle; 3) patients given a long-acting gonadotropin-releasing hormone (GnRH) agonist by subcutaneous injection; 4) patients with a positive pregnancy test; and 5) age <35 years. The exclusion criteria included: 1) women treated with antithrombotic drugs; 2) women with known coagulopathies; and 3) uncertain laboratory results and missing laboratory data. To analyze the relationship between the recovery time of patients with OHSS and the blood parameters such as leukocyte, hemoglobin, platelet, hematocrit, creatinine, total protein, albumin, prothrombin time (PT), activated partial thromboplastin time (APTT), thrombin time (TT), fibrinogen (Fib), D-dimer, and fibrinogen degradation products (FDPs) within 24 h after admission.

Treatment of OHSS usually involves fluid replacement to maintain intravascular perfusion and supportive care, such as low-molecular-weight dextrose and hydroxyethyl starch. The patient’s blood count, coagulation profile, electrolytes, creatinine, and albumin were observed. Depending on the patient’s condition, albumin was given intravenously, and anticoagulant drugs were given to patients with thrombotic tendency and hypercoagulable state to prevent thrombosis (18). Moreover, when the patient has large quantities of pleural and ascites, puncture and drainage were performed under ultrasound guidance. The details of the patients’ treatments are shown in **Table 1**.

**Table 1:** Conventional intervention for ovarian hyperstimulation syndrome (OHSS).

Treatment	*n*	%
Heparin	46	10.8
Albumin	321	75.7
Paracentesis		
Peritoneal puncture	219	51.7
Pleural puncture	52	12.3

A patient is clinically cured when the morning hematocrit was <40% and no obvious clinically relevant symptoms existed, such as abdominal distension, abdominal pain, and shortness of breath (19). On the other hand, ultrasound should indicate no pleural and abdominal effusion or a small amount of effusion, and the leukocyte count, creatinine, albumin, alanine transaminase (ALT), aspartate transaminase (AST), electrolytes, and other biochemical indicators should return to normal. The discharge criteria were considered the patients’ cure criteria.

### Laboratory Variables

The patients underwent basic blood routine, liver and kidney function, blood coagulation function, D-dimer, FDP, and other tests 24 h after admission. The levels of these parameters were measured using the Roche HP800 automatic biochemical analyzer and Sysmex series automatic blood analyzer.

### Controlled Ovarian Hyperstimulation Protocol

On the second to the third day of menstruation, the patients were given a long-acting GnRH agonist (Diphereline, 3.75 mg; Beaufour-Ipsen, Dreux, France) by subcutaneous injection. After 30 days, when the follicle-stimulating hormone (FSH) level was <5 IU/L, the LH level was <3 IU/L, and the antral follicle was nearly 5 mm in diameter, COH was initiated. We determined the individualized dosage of gonadotropin (Gn) (GONAL-f; Merck Serono, Darmstadt, Germany) according to the patient’s age, BMI, and ovarian reserve. The Gn dosage was maintained or adjusted according to the follicle growth and serum hormone levels during the course of the drug administration. When one dominant follicle was ≥20 mm in diameter and at least three dominant follicles were ≥17 mm in diameter, a trigger injection of hCG (recombinant hCG alpha for injection; Merck Serono) was administered on the same night. After 36–37 h of the trigger injection, we performed transvaginal oocyte retrieval; the luteal phase support was routinely given approximately 14 days after oocyte retrieval.

Two fresh cleavage embryos or one blastocyst was transferred on day 3 or 5 after egg retrieval. The transplant was cancelled if the patient was deemed at high risk of OHSS, the P level on the day of hCG was >3 ng/ml, or a uterine effusion was demonstrated.

### Statistical Analysis

All statistical analyses were conducted using IBM SPSS Statistics for Windows, version 26.0 (IBM Corp., Armonk, NY, USA). Descriptive variables were expressed as the mean and standard deviation (SD) if the data were normally distributed, as median and interquartile range (IQR) if the data were not normally distributed, or as frequency and percentage for nominal data. Spearman‘s correlation analysis was used to evaluate the associations between the variables of interest and the clinical outcomes. Multiple linear regression analysis was used when the outcomes were continuous variables. A bilateral *p*-value <0.05 was considered as significant.

## Results

**Figure 1:**
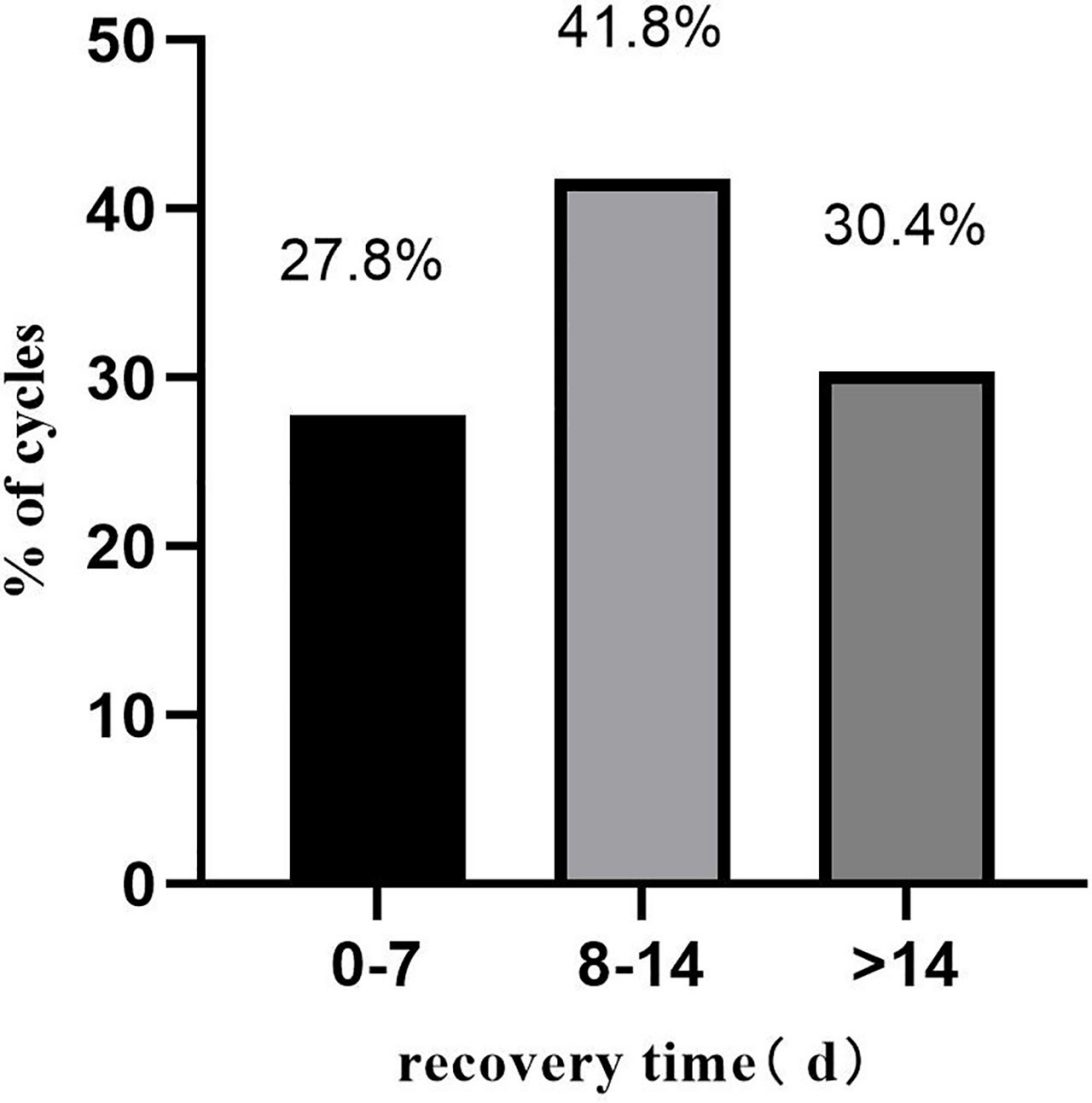
The recovery time distribution of the 424 OHSS patients.

A total of 424 pregnant patients who developed moderate to severe OHSS after IVF/ICSI treatment were included in this study. **Table 2** summarizes the basic information of these patients. The median recovery time of these patients was 11 days. A detailed distribution of the recovery times is shown in **Figure 1**.

**Table 2:** Baseline characteristics and cycle outcomes.

Variables	Measures
Age (years)	30 (27–31)
BMI (kg/m^2^)	20.8 (19.6–22.8)
AMH (μg/L)	4.27 (2.83–6.33)
Type of infertility (*n*, %)	
Primary infertility	241 (56.8%)
Secondary infertility	183 (43.2%)
Fertilization method (*n*, %)	
IVF	321 (75.7%)
ICSI	103 (24.3%)
PCOS (*n*, %)	51 (12%)
Gn use duration (days)	13 (12–14)
Gn dosage (IU)	1,662.5 (1,350–2,162.5)
Serum LH level on hCG day (mIU/ml)	0.91 (0.5–1.79)
Serum E2 level on hCG day (pg/ml)	3,437 (2,370.5–4,769)
Serum P level on hCG day (ng/ml)	0.81 (0.51–1.25)
No. of oocytes retrieved	13 (11–17)
Cleavage embryo or blastocyst (day 3 or 5)	
Day 3	315 (74.3%)
Day 5	109 (25.7%)
Severity of OHSS (*n*, %)	
Moderate	181 (42.7%)
Severe	243 (57.3)
Recovery time (d)	11 (7–15)
Pregnancy rate (n, %)	386 (91.0%)
Multiple pregnancy rate (n, %)	151 (35.6%)

BMI, body mass index; AMH, anti-müllerian hormone; IVF, in vitro fertilization; ICSI, intracytoplasmic sperm injection; PCOS, polycystic ovary syndrome; LH, luteinizing hormone; E2, estradiol; P, progesterone; OHSS, ovarian hyperstimulation syndrome; hCG, human chorionic gonadotropin.

The patients’ primary laboratory findings are shown in **Table 3**. Spearman‘s correlation coefficients were calculated between the patients’ recovery times and the laboratory indices examined on the day of their admission to the hospital. The levels of leukocytes, hemoglobin, platelets, hematocrit, creatinine, albumin, PT, Fib, TT, D-dimer, and FDPs were correlated with the time to healing. Leukocytes, hemoglobin, platelets, hematocrit, creatinine, PT, Fib, D-dimer, and FDP were positively correlated with recovery time. On the contrary, albumin and TT were negatively correlated with recovery time.

**Table 3:** Relationship between the laboratory findings and recovery time of patients with ovarian hyperstimulation syndrome (OHSS).

Laboratory findings	Normal range	Measures	Spearman‘s correlation	
			Correlation coefficient	*p*-value
Leukocyte (10^9^/L)	3.5–9.5	12.97 (10.6–15.91)	0.401	<0.001
Hemoglobin (g/L)	115–150	143.65 (135.25–153)	0.494	<0.001
Platelets (10^9^/L)	125–350	340.5 (293–386)	0.245	<0.001
Hematocrit (L/L)	0.35–0.45	0.429 (0.402–0.458)	0.478	<0.001
Creatinine (mmol/L)	20–115	58.75 (52–66.18)	0.352	<0.001
ALT (U/L)	0–40	26 (15.25–41)	−0.027	0.577
AST (U/L)	0–58	22.5 (17–32.75)	−0.071	0.146
Albumin (g/L)	35–55	37.5 (34.9–40.1)	−0.202	<0.001
PT (s)	8.8–13.6	10.3 (9.9–10.8)	0.151	0.002
APTT (s)	26–40	28.3 (26.6–30.2)	−0.075	0.125
Fib (g/L)	2–4	4.65 (4.3–5.32)	0.175	<0.001
TT (s)	10–18	12.6 (12.1–13.1)	−0.113	0.020
D-dimer (mg/L)	0–0.3	0.70 (0.52–0.95)	0.136	0.005
FDP (mg/L)	0–5	7.23 (5.19–10.54)	0.114	0.018

ALT, alanine aminotransferase; AST, aspartate transaminase; PT, prothrombin time; APTT, activated partial thromboplastin time; TT, thrombin time; Fib, fibrinogen; FDP, fibrinogen degradation products.

**Figure 2:**
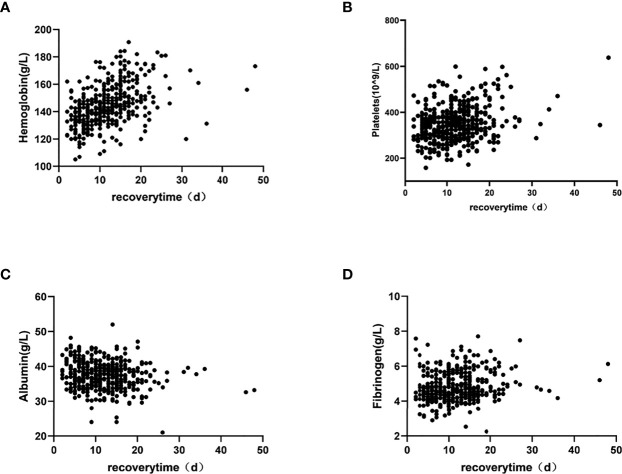
Relationship between the recovery time and hemoglobin, platelets, albumin and Fib.

All data with *p* < 0.05 in the above-mentioned correlation analysis and the high-risk factors affecting OHSS reported in the literature were included in the multiple linear regression. The results showed that PCOS, hemoglobin, platelets, albumin, and Fib significantly influenced the patients’ recovery times (*p* = 0.023, *p* < 0.001, *p* = 0.007, *p* < 0.001, and *p* = 0.019, respectively) (**Table 4**). Moreover, scatter diagrams were used to clearly describe the relationship between the recovery time and the levels of hemoglobin, platelets, albumin, and Fib (**Figure 2**). The remaining indicators were not highly correlated with the recovery time of patients.

**Table 4:** Multiple linear regression analysis of factors affecting recovery time.

Indexes	Unstandardized coefficients		Standardized coefficients	*t* value	*p*-value	Collinearity statistics	
	*B*	SE	*β*			Tolerance	VIF
Age (years)	−0.006	0.089	−0.003	−0.066	0.947	0.939	1.065
BMI (kg/m^2^)	−0.130	0.104	−0.055	−1.244	0.214	0.901	1.110
AMH (μg/L)	−0.086	0.082	−0.049	−1.051	0.294	0.805	1.242
PCOS	−1.988	0.871	−0.105	−2.281	0.023	0.821	1.218
Serum E2 level on hCG day (pg/ml)	0.000	0.000	0.005	0.102	0.919	0.877	1.141
No. of oocytes retrieved	0.073	0.059	0.061	1.245	0.214	0.733	1.365
Cleavage embryo or blastocyst	−0.314	0.582	−0.028	−0.540	0.589	0.669	1.495
Clinical pregnancy (singleton or multiple)	−0.190	0.607	−0.015	−0.312	0.755	0.779	1.284
Leukocyte (10^9^/L)	0.077	0.079	0.057	0.981	0.327	0.523	1.910
Hemoglobin (g/L)	0.146	0.024	0.349	6.213	<0.001	0.552	1.811
Platelets (10^9^/L)	0.010	0.004	0.129	2.690	0.007	0.752	1.329
Hematocrit (L/L)	0.039	0.069	0.025	0.570	0.569	0.939	1.065
Creatinine (mmol/L)	0.015	0.026	0.029	0.562	0.575	0.656	1.523
Albumin (g/L)	−0.239	0.067	−0.154	−3.587	<0.001	0.944	1.060
PT (s)	0.046	0.052	0.037	0.880	0.379	0.981	1.019
Fib (g/L)	0.835	0.356	0.113	2.348	0.019	0.755	1.324
TT (s)	−0.227	0.269	−0.043	−0.843	0.400	0.662	1.510
D-dimer (mg/L)	0.537	0.553	0.056	0.969	0.333	0.517	1.934
FDP (g/L)	−0.032	0.071	−0.024	−0.442	0.659	0.587	1.705

AMH, anti-müllerian hormone; PCOS, polycystic ovary syndrome; hCG, human chorionic gonadotropin; PT, prothrombin time; Fib, fibrinogen; TT, thrombin time; FDP, fibrinogen degradation product.

## Discussion

OHSS is a major iatrogenic complication that arises during the process of assisted reproductive technology (ART), affecting 0.5%–2% of IVF cycles. There are two distinct types of OHSS: early-onset OHSS occurs in response to the hCG trigger within 7 days of ovulation, while late-onset OHSS is caused by the rising hCG hormone levels produced by the placenta in conception cycles (10). The present study focused on the recovery time of conceived women with OHSS requiring hospitalization.

To date, there is no universally accepted definition of OHSS recovery. The definition used in this research was based on a previous study (19). Patients were discharged from the hospital when the morning hematocrit was <40% and no obvious clinically relevant symptoms existed, such as abdominal distension, abdominal pain, and shortness of breath. On the other hand, ultrasound indicating little pleural or abdominal effusion and biochemical abnormalities returning to normal were required. The median recovery time in our study population was 11 days. This is much longer than that reported in the literature, partly due to the particular population and our strict release criteria.

Current studies have revealed that the incidence of OHSS was associated with numerous clinical and laboratory parameters. The concentration of E2 on the day of hCG >4,500 pg/ml and the number of oocytes retrieved >15 are commonly proposed to be risk factors for OHSS (3). However, our results showed that the median concentration of E2 on the day of hCG was 3,437 pg/ml and that the median number of oocytes retrieved was 13. This is consistent with the study of Wiser et al. because the freeze-all strategy was carried out in women at high risk of OHSS after retrieval of oocytes (20). In addition, the concentration of E2 on the day of hCG is not the main reason for the late-onset OHSS: pregnancy remains the main cause (21). Despite scholars having proposed multiple pregnancy as a predictive factor for recovery time from OHSS, our research found no differences between patients with singleton and multiple pregnancies.

As demonstrated in **Table 4**, the presence of PCOS was negatively correlated with the recovery time of pregnant OHSS patients, which is in accordance with the result of Nouri et al. (19). PCOS appeared to be the major predisposing factor for OHSS in a large number of studies (2). The explanation might be that PCOS cases are known to produce three times more follicles and oocytes than do normal ovulation patients when stimulated according to similar protocols (6). However, the reason for the prolonged recovery time of patients with PCOS remains unknown. VEGF is thought to be the major mediator of OHSS (22). An increased expression of VEGF mRNA in women with PCOS has been reported, and this may be responsible for the prolonged recovery time (23). Nevertheless, this is completely unproven. Further investigations are necessary to confirm this hypothesis. It is our suggestion that more stringent embryo transfer criteria should be administered or that patients with PCOS undergo whole embryo cryopreservation.

The second predictive factor for recovery time is serum albumin. Hypoalbuminemia is associated with a significantly longer recovery time. The mechanisms underlying the potential effect of serum albumin on OHSS are unknown. Some studies have suggested that the binding properties of albumin are beneficial in neutralizing vascular permeability mediators (24). Other studies have shown that serum albumin could maintain the intravascular volume in the event of capillary leakage, thus avoiding hypovolemia and hemoconcentration (25). Therefore, the level of serum albumin may reflect the severity of OHSS.

Studies have demonstrated that the levels of leukocytes, platelets, hematocrit, Fib, D-dimer, and FDP in OHSS patients were higher in routine laboratory tests (26). As shown in **Table 3**, this is consistent with our study. Moreover, in multiple linear regression analysis, hemoglobin, platelets, and Fib were positively correlated with the recovery time, and the difference was statistically significant. There are two reasons for the changes in these blood-related parameters: stress and hemoconcentration (27). OHSS is a potentially lethal disease, the pathophysiological hallmark of which is massive extravascular exudate accumulation combined with profound intravascular volume depletion and hemoconcentration (28). The degree of hemoconcentration seems to have the best correlation with the severity of OHSS (29). The levels of hemoglobin, platelets, and Fib are the earliest and most sensitive indicators of changes in the blood and are also ideal predictors for recovery time from OHSS.

It seems that an elevation in circulating estrogens during ovulation induction causes a shift in the hemostatic balance in the direction of a procoagulable state. Fib is a conventional coagulation indicator that indicates the activation of the coagulation system. Coagulation causes an increased Fib consumption and promotes Fib synthesis in the body, resulting in increased plasma Fib levels. Thrombosis is the most serious complication of OHSS, leading to dysfunction of coagulation and fibrinolysis *in vivo*. Thrombosis causes secondary hyperfibrinolysis; as plasma D-dimer and FDP are degradation products of fibrinogen, their levels will therefore increase rapidly. Therefore, monitoring the levels of plasma Fib, D-dimer, and FDP can prompt clinical correction of hypercoagulable blood concentration. Heparin ameliorates the risk of thrombotic complications associated with OHSS and has become the recommended treatment protocol (30). VEGF plays a leading role in increasing vascular permeability, and a 5-kDa heparin fragment can inhibit VEGF-A-mediated angiogenesis (31). Tissue factor (TF) is also important in angiogenesis because it enhances the expression of VEGF-A, and heparin reduces this by inhibiting the release of TF (32).

This is the first study to comprehensively assess the coagulation function and recovery time of conceived women with moderate to severe OHSS. As a result of OHSS-specific symptoms and the costs of the treatment program, decreased quality of life and economic losses are inevitable. Therefore, recovery time should be a clinically important parameter, and our study is of great value. Simultaneously, this study has some limitations. We conducted this retrospective study without considering all confounding factors. Only patients receiving a particular ovulation induction program were included in our study. Moreover, differences in the dietary habits of patients during hospitalization have a certain impact on the recovery time from OHSS.

In general, the existence of PCOS and the levels of hemoglobin, platelets, albumin, and Fib may contribute to the prognostic evaluation of OHSS. In consideration of the particular study population and limitations, large-scale, multicenter, prospective studies are necessary to confirm our results.

## Data Availability Statement

The original contributions presented in the study are included in the article/supplementary material. Further inquiries can be directed to the corresponding author.

## Ethics Statement

The studies involving human participants were reviewed and approved by The First Affiliated Hospital of Zhengzhou University. Written informed consent for participation was not required for this study in accordance with the national legislation and the institutional requirements.

## Author Contributions

KH and JZ designed the research and guided the writing. YS collected and analyzed the data. KH and YS drafted the manuscript. G-ZC helped to collect and analyze data. HS contributed to the data analysis. All authors contributed to the article and approved the submitted version.

## Funding

This study was supported by the National Natural Science Foundation of China (nos. 81701448 and 82071649).

## Conflict of Interest

The authors declare that the research was conducted in the absence of any commercial or financial relationships that could be construed as a potential conflict of interest.

## Publisher’s Note

All claims expressed in this article are solely those of the authors and do not necessarily represent those of their affiliated organizations, or those of the publisher, the editors and the reviewers. Any product that may be evaluated in this article, or claim that may be made by its manufacturer, is not guaranteed or endorsed by the publisher.
